# Perceived risk for screen-detectable cancers among american indian adults in the zuni pueblo, USA: Insights and implications for intervention programs

**DOI:** 10.1016/j.pmedr.2024.102950

**Published:** 2024-12-17

**Authors:** Deborah Kanda, Kate Cartwright, V. Shane Pankratz, Judith Sheche, Mikaela Kosich, Nicholas Edwardson, Samantha Leekity, Shiraz I. Mishra

**Affiliations:** a University of New Mexico Comprehensive Cancer Center, University of New Mexico Health Sciences Center, USA; b University of New Mexico, School of Public Administration, USA; c Department of Internal Medicine and University of New Mexico Comprehensive Cancer Center, University of New Mexico Health Sciences Center, USA; d was affiliated with the University of New Mexico Health Sciences Center and its Comprehensive Cancer Center at the time of this research, USA

**Keywords:** Perceived cancer risk, American Indians, Health equity, Community-partnered participatory research, Rural

## Abstract

**Background:**

Perceptions of disease risk play an important role in adopting healthy behaviors. The main objective of this study is to examine factors associated with high perceived cancer risk among Zuni Adults in New Mexico, USA.

**Methods:**

We used data from a survey conducted in Zuni Pueblo from October 2020 to April 2021. Our analysis included 254 adults ages 21–75 years without a reported personal cancer history. Perceived cancer risk was determined from the question: “Compared to other people your age, how likely do you think it is that you could get cancer?” and ordinal logistic regression analyses were used to identify factors associated with high perceptions of cancer risk.

**Results:**

Overall, 35 %, 27 %, and 38 % of respondents reported perceived cancer risks that were lower than, about the same as, and higher than those of other people their age, respectively. From bivariate analysis, factors associated with high perceived cancer risk included: positive family cancer history (odds ratio [OR] = 1.95; 95 % confidence interval [CI]: 1.23–3.11), higher knowledge of cancer risk factors (OR = 1.45; CI: 1.09–1.93), higher education (OR = 1.46; CI: 1.16–1.84), and higher body mass index (OR = 1.44; CI: 1.07–1.94). In multivariable analysis, family cancer history (OR = 1.81; CI: 1.10–2.99), knowledge of risk factors (OR = 1.38; CI: 1.03–1.86), and education (OR = 1.81; CI: 1.10–2.96) remained statistically significant.

**Conclusion:**

Our findings provide important insights on perceptions of cancer risk in this community, and have important implications for developing effective, culturally relevant interventions in this community.

## Highlights

• We examined perceived cancer risk in a population-based sample of American Indians.
• A high proportion of participants reported high perceptions of cancer risk.
• Risk predictors were family history, knowledge of risk factors and education.
• Study provides important insights for developing culturally relevant interventions.

## 1 Introduction

American Indian and Alaska Natives (AIAN) have higher overall cancer incidence compared to non-Hispanic White individuals (NHW) and this inequity in incidence rates carries over to cancer mortality as well (Kratzer et al., 2023). Recent data suggest that compared to NHW, AIAN individuals experience 2 % greater cancer incidence overall, and an 18 % higher rate of cancer mortality (Kratzer et al., 2023). Similarly, a report compiled by the American Cancer Society on cancer statistics from 2016 to 2020 shows that for all cancer sites combined, AIAN individuals have higher mortality rates than any other racial group (American Cancer Society, 2023a). These disparities could be reduced by increasing access to high-quality cancer prevention services, and adopting health-protective behaviors and preventive screening (American Cancer Society, 2023b). Compared to other race/ethnic groups, AIAN individuals have some of the lowest screening rates for the three screen-detectable (i.e., breast, cervical, and colorectal) cancers (Sabatino et al., 2022). Data from a national survey showed that AIAN individuals are less likely than NHW to be up-to-date for breast (64.3 % versus 76 % respectively), cervical (75.6 % versus 77.9 %), and colorectal cancer screening (62.8 % versus 69.8 %) (Sabatino et al., 2022). For AIAN individuals, cancer is diagnosed at more advanced stages of the disease, which is associated with higher rates of mortality (Giaquinto et al., 2022; Roubidoux et al., 2022). If cancer screenings among AIAN individuals can be conducted earlier and consistently, in compliance with cancer screening guidelines, there is an opportunity to identify cancer at early stages and reduce mortality rates (Melkonian et al., 2019; Roubidoux et al., 2022).

While there are many factors that influence cancer screening behaviors, one body of literature examines how individuals' perceived risk of cancer influences their behaviors to seek screening. Several health behavior models including the Health Belief Model (Janz and Becker, 1984), the Precaution Adoption Process Model (Weinstein, 1988), and Protection Motivation Theory (Rogers, 1975), propose that an individual's perception of disease risk is an important predictor of adopting preventive health behaviors. Research suggests that there are racial/ethnic differences in cancer risk perceptions, with non-White individuals reporting lower perceived risk compared to NHW individuals (Davis et al., 2013; Orom et al., 2010). Understanding cancer risk perception of AIAN individuals, as well as the determinants of such perceptions, is important for developing and implementing culturally appropriate interventions that will reduce cancer disparities.

The differences in risk perceptions for developing screen-detectable cancers in different race/ethnic groups may imply that social and ethnic factors may be associated with cancer risk perception, and therefore may be a potential barrier to adopting preventive screenings. Very few studies have examined perception of cancer risk among American Indians. (Gonzales et al., 2010) provided one of the early investigations of factors associated with perceived cancer risk among American Indians. Their study focused on Hopi Tribal members residing in northeastern Arizona and provided insights on this topic from this specific Tribe. Many population-health studies report on AIAN as one group, which helps give broad understanding about the severity of disparities, but the AIAN population is incredibly diverse and these studies cannot address Tribe-specific disparities or needs. (Safi et al., 2022) conducted a qualitative study among 51 Zuni tribal members to understand their knowledge and perceptions about cancer in general. While their study provided great insights on how the Zuni people conceptualize cancer in general, it does not capture their perceptions on cancer risk (related to screen-detectable cancers). To the best of our knowledge, no studies have quantitatively examined the perceptions of cancer risk within the Zuni Tribe. The goal of this study is to examine perceived cancer risk (related to screen-detectable cancers, i.e., breast, cervical, and colorectal), including the associations between various social determinants of health as well as cancer screening behaviors among American Indian adults in the Zuni Pueblo. The results from this study will provide important, community-specific insights on perceptions of cancer risk as related to three screen-detectable cancers among AIAN individuals, thereby contributing to the limited research on risk perceptions in this population, and will also provide important implications for developing effective, culturally relevant interventions.

## 2 Methods

### 2.1 Research setting

Data for this cross-sectional study are from a community-engaged cancer control survey conducted in Zuni Pueblo. The Pueblo is located in a rural region of western New Mexico. Zuni Indians have lived in the present Pueblo for almost 400 years and differ culturally from other American Indian tribes (Stidley et al., 2003). There are about 11,000 Zuni, and an estimated 7000 full-time residents in the Pueblo with median age of 33 years (U.S. Census Bureau, 2022). Researchers at the University of New Mexico Health Sciences Center (UNM HSC) in collaboration with Zuni Pueblo formed the Zuni Health Initiative (ZHI), to conduct community-based participatory research for planning and implementing health promotive interventions in the Pueblo (Shah et al., 2014). This study received research approval from the Zuni Pueblo Tribal Council, the Southwest Tribal Institutional Review Board (IRB) and the UNM HSC IRB.

### 2.2 Sampling strategy and eligibility criteria

The sampling strategy has been described previously (Cartwright et al., 2022). In brief, all the streets in Zuni Pueblo were enumerated and some streets were selected at random. Survey recruitment flyers were then distributed by ZHI staff in all households of selected streets. Also, flyers were distributed at local businesses and high traffic locations and public radio announcements were made on the community radio station. Participants were deemed eligible if: they self-identify as American Indian, belong to Zuni tribe or are married to a Zuni tribal member, and meet the age and gender requirements for the age/gender-specific survey, which corresponded to the United States Preventive Services Task Force-recommended age-eligibility for particular cancer screenings (i.e., either between 21 and 75 years old for females (breast, cervical, & colon-rectum) or 50–75 years old for males (colon-rectum only).

### 2.3 Survey implementation

The ZHI staff administered surveys to eligible participants from October 2020 through April 2021. Overall, 280 participants completed the surveys: 61 were completed by women ages 21–49, 110 by women ages 50–75, and 109 by men ages 50–75. The survey documented age/gender-specific cancer-related screening patterns, attitudes, beliefs, and knowledge regarding cancers of the breast, cervix, and colon-rectum. Women ages 21–49 were surveyed about cervical cancer; women ages 50–75 about breast, cervical, and colorectal cancer; and men ages 50–75 about colorectal cancer. The survey varied in length based on age and gender. Due to COVID-19 precautions, surveys were administered over the phone. All participants provided informed consent and they were given a merchandise card to compensate for their time. For this analysis, we excluded 24 individuals who reported a positive personal history of cancer, and 2 participants who were missing data on the perceived risk question, leaving a final sample size of 254.

### 2.4 Dependent variable

Perceived risk of developing cancer was measured in a slightly different manner among the three survey groups. Women ages 50–75 years were asked “compared to other people your age, how likely do you think it is that you could get cancer?” A similar question was used for women ages 21–49 but specific to cervical cancer: “compared to other people your age, how likely do you think it is that you could get cervical cancer?” Men ages 50–75 were asked the question: “compared to other people your age, how likely do you think it is that you could get colorectal cancer?” Response options to these questions were on a 5-point Likert scale and included: “much less likely”, “less likely”, “no difference”, “more likely”, and “much more likely”.

### 2.5 Independent variables

Selection of variables for study drew support from extant literature (Hay et al., 2006; Honda and Neugut, 2004; Lucas-Wright et al., 2014; Tilburt et al., 2011) on factors influencing cancer risk perception and these included: socio-demographic variables (age, sex, marital status, and education); family history of cancer; modifiable cancer risk factors (obesity measured by the body mass index [BMI] and exercise frequency). BMI was computed based on participants' reported weight and height values and categorized following the definition by the Centers for Disease Control and Prevention as normal/underweight (< 25 kg/m2), overweight (25.0–29.9 kg/m2), and obese (≥ 30 kg/m2) (Centers for Disease Control and Prevention, 2022). Exercise frequency was measured with the question: “How often do you exercise?”, measured as more than 3 times per week, one to 3 times per week, and I do not exercise regularly. Fatalistic attitude about cancer prevention was measured based on agreement or disagreement with the statement - “There is not much you can do to lower your chances of getting cancer.” Knowledge of cancer risk factors was measured using an aggregate of cancer-specific (breast, cervical, and colorectal cancers) risk factors. Specific items in the knowledge questions have been described previously (Cartwright et al., 2023a; Cartwright et al., 2023b; Edwardson et al., 2023). A knowledge score was created by counting correct responses to questions about cancer-specific risk factors (21 items for breast cancer, 18 for cervical, and 15 for colorectal cancer) by survey group and subsequently, the score was categorized into terciles and labeled as low, medium, or high. Women 21–49 years completed knowledge questions on cervical cancer only, and had total scores ranging from 4 to 13 out of 18, with tercile cut-off points of 8 and 10 at the 33rd and 66th percentiles respectively. Men 50–75 completed knowledge questions on colorectal cancer only and had scores ranging from 4 to 14 out of 15, with tercile cut-off points of 8 and 10 at 33rd and 66th percentiles respectively. While Women 50–75 completed questions on breast, cervical and colorectal cancers, and had scores ranging from 19 to 39 out of 54, with tercile cut-off points of 28 and 31 at the 33rd and 66th percentiles respectively.

We also looked at associations between screening behavior patterns for cancers of the breast, cervix, and colon-rectum and perceived risk for developing these screen-detectable cancers. Breast cancer screening questions were specific to women ages 50–75 years and for this, we included the variable “Have you ever had a mammogram?” measured as yes or no. Cervical cancer screening questions targeted all the surveyed women ages 21–75 years and included the variable “Ever had a cervical cancer screening (specifically a Pap or HPV test)?” measured as yes or no. Finally, colorectal cancer screening questions were directed to men and women between 50 and 75 years, which included the variables “Ever had a stool blood test?” and “Ever had a colonoscopy?” both of which were measured as yes or no. Due to disruption of preventive services, including cancer screenings, as a result of COVID-19, our analysis focuses on ever being screened for the specific cancer type rather than adherence to any specific cancer screening guidelines.

### 2.6 Statistical analysis

We conducted descriptive and bivariate analyses to summarize demographic and survey responses from the study participants, and to determine associations between the independent variables as well as cancer screening behavior patterns and perceived risk for developing screen-detectable cancers. Perceived cancer risk was measured on a 5-point Likert scale. Prior to analysis, we modified the dependent variable from a 5- to 3-point scale by coding “much less likely” and “less likely” categories as 1(low risk); no difference as 2 (medium risk); and “more likely” and “much more likely” as 3 (high risk), because of sparse cell counts in extreme levels of the dependent variable (i.e. levels 1 and 5) when cross-classified with some of the covariates.

We used ordinal logistic regression analyses to identify factors associated with higher perceptions of risk for developing screen-detectable cancers, as this approach is well suited to modeling relationships using an ordered dependent variable; important information would be discarded when the ordinality of the dependent variable is not fully exploited and the variable is collapsed into two levels for binary logistic regression (Scott et al., 1997). A commonly used ordinal regression model is the cumulative logit (Walker and Duncan, 1967) also known as proportional odds model (POM) (McCullagh, 1980). We fitted a POM to the 3-level perceived cancer risk variable and checked the POM assumption using both score tests and graphical methods (Peterson and Harrell Jr, 1990). A POM estimates the cumulative probability (or cumulative odds) that a response is classified at or below a given category. In our case, we estimated the odds of reporting medium to high risk (versus low risk). Multicollinearity was tested by examining inter-correlations of independent variables; all variance inflation factor values were well below 10, with the largest being 1.31, indicating no multicollinearity. Results from the models (univariable and multivariable) were reported as odds ratios with 95 % confidence intervals and statistical significance was defined at *p* < 0.05. Statistical analyses were done using SAS 9.4 (Cary, NC).

## 3 Results

### 3.1 Descriptive statistics

Overall, 35 %, 27 %, and 38 % of participants respectively reported low, medium, and high perceptions of risk for developing screen-detectable cancers. Table 1 shows the distribution of participants' characteristics by cancer risk perception and their bivariate relationships. Distributions of cancer risk perceptions varied by education level, family history of cancer, and exercise frequency. A greater proportion of individuals with high perceived risk for developing screen-detectable cancers were of higher education level, 58 % versus 42 % (*p* = 0.006). Similarly, individuals that affirmed a positive family cancer history were more likely to report medium and high perceptions of cancer risk: 51 % and 70 % respectively (*p* = 0.009). Of the 116 respondents who reported exercising more than three times per week 49 (42 %), 23 (20 %), and 44 (38 %) reported low, medium, and high perceived cancer risk, respectively. Table 1 also shows distributions of various cancer screening behaviors (breast, cervical, and colorectal cancers) by risk perceptions. There were no marked differences in cancer screening behavior and the perceived cancer risk categories.

**Table 1:** Descriptive characteristics of the study sample along with their associations to perceived risk for screen-detectable cancers among American Indian Adults in the Zuni Pueblo (*N* = 254): 2020 to 2021.

	Total, N (%)	Perceived cancer risk			*P*-value ⁎
		Low, n (%)	Medium, n (%)	High, n (%)	
Overall	254 (100)	89 (35)	69 (27)	96 (38)	–
Age, Median (interquartile range)	54 (50, 62)	54 (50, 64)	54 (49, 58)	54 (49, 62)	0.6
Survey group					0.3
Men 50–75 years	96 (38)	41 (46)	21 (30)	34 (35)	
Women 21–49 years	60 (24)	19 (21)	17 (25)	24 (25)	
Women 50–75 years	98 (39)	29 (33)	31 (45)	38 (40)	
Sex					0.11
Men	96 (38)	41 (46)	21 (30)	34 (35)	
Women	158 (62)	48 (54)	48 (70)	62 (65)	
Married/Partner					0.09
Live in Partner	123 (49)	36 (40)	40 (58)	47 (49)	
Single	130 (51)	53 (60)	29 (42)	48 (51)	
(Missing)	1	0	0	1	
Education level					0.006
≤ High school or GED	135 (53)	58 (65)	37 (54)	40 (42)	
> High school	119 (47)	31 (35)	32 (46)	56 (58)	
Income group					0.5
<$10,000	113 (45)	45 (52)	31 (46)	37 (39)	
$10,000 - $19,999	68 (27)	23 (26)	18 (26)	27 (29)	
>$20,000	68 (27)	19 (22)	19 (28)	30 (32)	
(Missing)	5	2	1	2	
Regular provider					0.5
No	107 (42)	37 (42)	33 (49)	37 (39)	
Yes	145 (58)	52 (58)	35 (51)	58 (61)	
(Missing)	2	0	1	1	
BMI					0.12
Normal range/underweight	66 (26)	31 (35)	18 (26)	17 (18)	
Overweight	101 (40)	33 (37)	26 (38)	42 (44)	
Obesity	87 (34)	25 (28)	25 (36)	37 (39)	
Exercise frequency					0.05
Daily or more than 3 times per week	116 (46)	49 (55)	23 (33)	44 (46)	
One to 3 times per week	83 (33)	24 (27)	31 (45)	28 (29)	
You do not exercise regularly	55 (22)	16 (18)	15 (22)	24 (25)	
Family cancer history					0.009
No	107 (42)	45 (51)	33 (49)	29 (30)	
Yes	146 (58)	44 (49)	35 (51)	67 (70)	
(Missing)	1	0	1	0	
Knowledge of risk factors					0.12
1st tercile	76 (30)	35 (39)	19 (28)	22 (23)	
2nd tercile	83 (33)	27 (30)	25 (36)	31 (32)	
3rd tercile	95 (37)	27 (30)	25 (36)	43 (45)	
Fatalistic attitude					0.8
Yes	94 (37)	34 (38)	23 (33)	37 (39)	
No	160 (63)	55 (62)	46 (67)	59 (61)	
*Cancer Screening Behavior*					
*Breast cancer screening (women 50–75 years, N = 98)*					
Ever had a mammogram	84 (86)	24 (83)	25 (81)	35 (92)	0.4
*Cervical cancer screening (women 21–75 years, N = 158)*					
Ever had a test to check for cervical cancer	117 (75)	33 (69)	38 (79)	46 (75)	0.5
(Missing)	1	0	0	1	
*Colorectal cancer screening (men and women ages 50–75 years, N = 194)*					
Ever had a stool blood test	80 (41)	32 (46)	19 (37)	29 (41)	0.6
(Missing)	1	0	0	1	
Ever had a colonoscopy	61 (31)	26 (37)	15 (29)	20 (28)	0.4

Abbreviations: BMI, body mass index categorized following the definition by the Centers for Disease Control and Prevention.

⁎ Kruskal-Wallis rank sum test for continuous variables; Pearson's Chi-squared test for categorical variables; Cochran-Mantel-Haenszel test for trend for ordinal variables.

### 3.2 Ordinal logistic regression analysis

Table 2 shows both univariable (unadjusted) and multivariable (adjusted) regression models. In univariable models, education, BMI, family cancer history, and knowledge of cancer risk factors were significantly associated with high perceptions of risk for developing screen-detectable cancers. Respondents with more than high school education had 1.46 (odds ratio [OR] = 1.46; 95 % confidence interval [CI]: 1.16–1.84) times higher odds of perceiving themselves at higher risk of developing cancer compared to those with only high school or lower education level. Similarly, individuals with higher BMI (OR = 1.44; CI: 1.07–1.94), positive family cancer history (OR = 1.95; CI: 1.23–3.11), and higher knowledge of cancer risk factors (OR = 1.45; CI: 1.09–1.93) were more likely to report high perceptions of cancer risk. In multivariable analysis, higher education level (OR = 1.81; CI: 1.10–2.96), positive family cancer history (OR = 1.81; CI: 1.10–2.99), and higher knowledge of cancer risk factors (OR = 1.38; CI: 1.03–1.86), remained statistically significant.

**Table 2:** Unadjusted and adjusted ordinal logistic regression models testing the association between independent variables and perceived risk for screen-detectable cancers among American Indian Adults in the Zuni Pueblo, USA (N = 254): 2020 to 2021.

	Unadjusted model	Adjusted model
	Odds Ratio (95 % CI)	Odds Ratio (95 % CI)
Age	1.0 (0.98, 1.02)	1.0 (0.98, 1.03)
Sex		
Men	Reference	Reference
Women	1.4 (0.89, 2.29)	1.1 (0.64, 1.93)
Married/Partner		
Live in Partner	Reference	Reference
Single	0.8 (0.48, 1.20)	0.8 (0.51, 1.35)
Education		
≤ High school or GED	Reference	Reference
> High school	1.5 (1.16, 1.84)	1.8 (1.10, 2.96)
Have a regular provider		
No	Reference	Reference
Yes	1.1 (0.69, 1.74)	0.8 (0.48, 1.35)
BMI	1.4 (1.07, 1.94)	1.3 (0.94, 1.80)
Exercise frequency	0.8 (0.61, 1.09)	0.9 (0.67, 1.27)
Family cancer history		
No	Reference	Reference
Yes	2.0 (1.23, 3.11)	1.8 (1.10, 2.99)
Knowledge of risk factors	1.5 (1.09, 1.93)	1.4 (1.03, 1.86)
Fatalistic attitude		
No	Reference	Reference
Yes	1.0 (0.63, 1.63)	1.2 (0.70, 1.90)

Abbreviations: OR, Odds Ratio; CI, Confidence Interval; BMI, body mass index.

Score test for the proportional odds assumption: Chi-square = 12.17, df = 10, P-value = 0.27.

The variables: BMI, exercise frequency, and knowledge of risk factors were treated as continuous variables to allow for ordinal comparisons.

## 4 Discussion

This study examined perceptions of risk for developing screen-detectable (i.e., breast, cervical, and colorectal) cancers in a sample of Zuni tribal members aged 21 to 75 years without reported personal cancer history. Our results show that about 38 % of participants believed that their risk of developing cancer was more than that for others their age. This proportion is higher than the 29 % observed in a random sample of Hopi tribal members having moderate to high perceptions of cancer risk (Gonzales et al., 2010) and greatly exceeds the 8.7 % observed in the general US population (Honda and Neugut, 2004), and may be indicative of a general pessimistic bias regarding cancer risk in this population.

Our results provide evidence that there are statistically significant differences in the factors associated with high perceived cancer risk. We found that respondents with higher education attainment, higher BMI, higher knowledge of cancer risk factors, and positive family history of cancer reported increased perceived risk for developing screen-detectable cancers compared to respondents without these factors. The findings that greater knowledge of cancer risk factors and higher educational attainment are associated with higher perceived cancer risk support earlier findings (Glanz et al., 1999; Lucas-Wright et al., 2014; Orom et al., 2010) which suggest that education should build capacity for heightened perception of individual cancer risk. Similarly, the association between family history of cancer and perceived cancer risk is consistent with other studies (Amuta et al., 2017; Gonzales et al., 2010; Honda and Neugut, 2004; Lucas-Wright et al., 2014), and a rationale is that those who have a family member experience cancer may internalize that information into their own perception of cancer risk (Gonzales et al., 2010). Lastly, the finding of higher BMI being associated with higher perceived cancer risk may differ from those of other factors. For instance, individuals who are overweight or obese may be given more information about long-term health effects more regularly than those who are not overweight.

### 4.1 Implications

Our findings provide important directions for developing future cancer screening and risk communication messages. First, the high level of pessimism bias seen in this population underscores the need for messages that dispel fear and are targeted towards empowering Zuni Indians on their ability to prevent cancer and reduce risk susceptibility as fear has been linked to pessimism bias (Powe and Finnie, 2003; Safi et al., 2022; Sheche et al., 2024). The qualitative study on how Zuni people conceptualize cancer in general reported themes that centered around the uncontrollable, unpredictable, and inevitably fatal nature of cancer (Mishra et al., 2023; Safi et al., 2022). Indeed, this belief about cancer is not unique to Zuni Tribe, previous research among the Navajo reflects similar perceptions about cancer (Bea et al., 2020; Csordas, 1989). Such fatalistic perceptions of cancer may hamper participation in programs that raise awareness of cancer risk factors and encourage preventive screening. As such, underscores the need for messages that address the underlying fear of cancer while highlighting the different ways that Tribal members think about cancer prevention and treatment. It is crucial that the development, testing, and refinement of empowering messages to promote cancer screening are centered around community input to better assure cultural specificity.

Second, because increased knowledge of cancer risk factors and education is associated with higher risk perceptions, it is important that when we implement interventions that aim to improve cancer knowledge and cancer screening, we need to support people with new higher perceived risk to feel empowered towards adopting healthier behaviors. Specifically, if our cancer education interventions increase perceived cancer risk resulting in increased fatalism in some individuals, we must have strategies in place to support this group. Cancer education interventions should identify increased fatalism as soon as possible. These interventions should have messages of hope and empowerment emphasizing that many cancers (especially the screen-detectable cancer such as cancers of breast, cervix, and colon-rectum) are preventable or highly treatable when identified early and that the impact of treatment is the least invasive when identified at early stages (Sheche et al., 2024).

Patient health navigators could be trained to do specialized health coaching with empowering messages. Interventions could include culturally-specific and appropriate stories of survivors and their quality of life. In order to be the most effective at addressing AIAN cancer health inequities, our interventions should lead to guideline-concordant cancer screening for all. Importantly, anyone working to improve cancer screening rates must develop strategies to address fatalism while at the same time honoring cultural beliefs and norms about death and dying. As interventions are implemented to improve cancer knowledge including information which will influence individual cancer risk perceptions, researchers and healthcare workers should have measures in place to identify individuals who experience higher degrees of fatalism along with higher degrees of cancer knowledge. Strategies should be developed to address fatalism so individuals may be better supported to pursue preventive health screenings. These strategies need to be culturally tailored to specific Tribal beliefs, norms, and priorities.

Third, it is important to recognize that the concept of family in many American Indian communities can be quite broad and risk internalization processes may go beyond immediate family members to extended families, clan relatives or even non-biologically related individuals (Gonzales et al., 2010). As such, health care providers need to understand relevant community dynamics and use appropriate kinship terms when collecting information on family histories to ensure that the relevant information is about biologically related family. Additional strategy to circumvent cultural taboos limiting discussions around cancer within the family setting is needed.

Lastly, it is crucial that educational interventions also address individuals who are overweight or obese and feel that they have elevated cancer risk, informing them that any risk for cancer can be lowered by altering behaviors that affect their weight, including an adequate level of physical activity and maintaining a healthy diet (Silverman et al., 2017).

### 4.2 Limitations, strength, and future directions

There are some limitations of this study to be considered. First, this study was cross-sectional and therefore causal claims cannot be established. Additionally, respondents self-selected to complete the survey which could lead to selection bias thereby limiting the generalizability of our findings. Also, our data is based on self-report and could suffer from inherent biases such as social desirability bias. Furthermore, because of the uniqueness of Zuni Pueblo's community and culture, our results are likely not generalizable to all AIAN communities. However, this study contributes to the limited research on cancer risk perceptions among AIAN population particularly among Zuni Tribal members. There is a need for research to understand how perceived cancer risk, especially for screen-detectable cancers, is associated with cancer screening behaviors for specific Tribes in order to develop effective cancer screening programs. Future work should examine how perceived cancer risk is related to specific outcomes of health empowerment and health fatalism regarding cancer screening and prevention efforts.

## Funding

This research was supported by the National Cancer Institute of the National Institutes of Health Grant R01CA192967 (Mishra PI), UNM Comprehensive Cancer Center (UNMCCC) Support Grant NIH/NCI P30CA118100 (Sanchez, PI), supplements to the UNMCCC Support Grant NIH/NCI P30CA118100 (Sanchez PI; Mishra, PD), UNMCCC Institutional Pilot Awards (PP-U1418-RS, PP-U1402-CaC, Mishra, PI), and the Institutional Development Award (IDeA) from the NIH/NIGMS P20GM103451 under the New Mexico IDeA Networks of Biomedical Research (NM-INBRE) Developmental Research Project Program (Mishra, PI of the Developmental Research Project).

## Ethics Approval

This study received approval from the Zuni Pueblo Tribal Council, the Southwest Tribal Institutional Review Board (IRB), and the University of New Mexico Health Sciences Center IRB.

## CRediT authorship contribution statement

**Deborah Kanda:** Writing – original draft, Methodology, Conceptualization. **Kate Cartwright:** Writing – review & editing, Writing – original draft, Conceptualization. **V. Shane Pankratz:** Writing – review & editing, Methodology, Formal analysis. **Judith Sheche:** Writing – review & editing, Resources. **Mikaela Kosich:** Writing – review & editing, Data curation. **Nicholas Edwardson:** Writing – review & editing. **Samantha Leekity:** Writing – review & editing, Resources. **Shiraz I. Mishra:** Writing – review & editing, Supervision, Resources, Funding acquisition, Conceptualization.

## Declaration of competing interest

The authors declare that they have no known competing financial interests or personal relationships that could have appeared to influence the work reported in this paper.

## Data Availability

The authors do not have permission to share data.
